# Which is the best laser for ureteroscopy? A Comprehensive Review

**DOI:** 10.1590/S1677-5538.IBJU.2026.0195

**Published:** 2026-04-30

**Authors:** Vinícius Ravena Lourenço, João Pedro Leal Puppin, Fábio Carvalho Vicentini, Alexandre Danilovic, Giovanni Scala Marchini, Fábio César Miranda Torricelli, Carlos Alfredo Battagello, Rodrigo Perrella, Anderson Pellanda, William Carlos Nahas, Eduardo Mazzucchi

**Affiliations:** 1 Universidade de São Paulo Faculdade de Medicina Departamento de Urologia São Paulo SP Brasil Departamento de Urologia, Faculdade de Medicina, Universidade de São Paulo - FMUSP, SP, São Paulo, Brasil; 2 Universidade de São Paulo Faculdade de Medicina Departamento de Cirurgia São Paulo SP Brasil Departamento de Cirurgia, Faculdade de Medicina, Universidade de São Paulo - FMUSP, SP, São Paulo, Brasil

**Keywords:** Ureteral Calculi, Ureteroscopy, Ureter

## Abstract

**Purpose:**

The landscape of ureteroscopy has been transformed by rapid advancements in laser technology. The transition from the standard Holmium:YAG (Ho:YAG) to Thulium Fiber Laser (TFL) and emerging hybrid technologies (Magneto/Tm:YAG) creates decisional uncertainty. This review analyzes the physical principles, safety, and clinical outcomes of available lasers, proposing a context-based decision-making framework.

**Materials and methods:**

A literature review using PubMed and Embase (2014-2025) yielded 1,902 records. Search terms included "holmium laser nephrolithotripsy," "thulium fiber laser," and "ureteroscopy laser." Fourteen seminal high-quality studies were selected for expert narrative synthesis, focusing on ablation efficiency, stone-free rates (SFR), complications, and cost-effectiveness.

**Results:**

Literature heterogeneity, particularly inconsistent SFR definitions and imaging modalities, challenges comparative analysis. Clinically, TFL demonstrates superior ablation speeds and dusting capabilities compared to standard Ho:YAG, with equivalent safety. However, High-Power Ho:YAG remains unmatched in versatility for complex stones. Emerging pulsed Tm:YAG and Magneto Cyber Ho systems theoretically combine peak power with dusting efficiency but require further validation. Economically, TFL and High-Power systems demand significantly higher capital investment than Low-Power alternatives.

**Conclusions:**

No universally superior laser exists; selection must be guided by institutional context. We propose a four-scenario framework: (1) Low-Power Ho:YAG is ideal for cost-sensitive, low-volume centers; (2) High-Power Ho:YAG is the standard for high-volume centers treating complex cases; (3) TFL is the choice for maximizing operative efficiency and dusting; and (4) Emerging Hybrid Technologies suit academic centers driving innovation. Future studies must prioritize standardized reporting to solidify the evidence base.

## INTRODUCTION

In ureteroscopy, lasers have gained significant prominence. However, the diversity of laser types, characteristics, and applications can create decisional uncertainty. This review provides a comprehensive introduction to laser technology while updating specialists with recent advancements and insights in the field.

Since 1995, the Holmium Aluminum Garnet (Ho:YAG) laser has revolutionized lithotripsy, rapidly becoming the gold standard for urinary stone treatment due to its ability to fragment all stone types, high efficiency, and minimal tissue penetration (1). Building on the success of Ho:YAG, recent alternative laser technologies have emerged to enhance endoscopic lithotripsy performance, including thulium-based systems that demonstrate improved ablation characteristics and reduced stone retropulsion (2-4). Concurrent advancements in optical fiber delivery systems and pulse modulation technologies have further optimized energy transmission, improved safety profiles, and increased overall ablation efficiency (2).

These technological developments have expanded the available options for urologists. Indeed, recent literature continuously highlights how the refinement of retrograde intrarenal surgery (RIRS) techniques and equipment directly impacts clinical outcomes and patient safety (5, 6). The aim of this article is to present a literature review of the available options and provide a decision-making framework to assist urologists in selecting the appropriate device based on clinical reality.

While previous reviews have extensively compared standard Ho:YAG and TFL, literature lacks a pragmatic guide incorporating the newest hybrid and pulsed solid-state technologies (such as pulsed Tm:YAG and Magneto). Therefore, the novelty of this review lies in moving beyond a simple physical and clinical comparison. We provide an updated economic overview and propose a novel, context-based decision-making framework. This approach is highly important for the current urological literature as it assists surgeons, department heads, and healthcare managers in tailoring laser acquisition to their specific institutional realities, balancing budget constraints, surgical volume, and clinical needs (7).

## MATERIALS AND METHODS

A comprehensive review was conducted on PubMed and Embase. The search terms included: "holmium laser nephrolithotripsy", "thulium fiber laser lithotripsy", "thulium YAG laser lithotripsy", and "ureteroscopy laser". Articles published from 2014 to 2025 were included. Additionally, seminal works deemed fundamental to the topic were incorporated regardless of their publication date or the aforementioned inclusion criteria.

**Figure 1:**
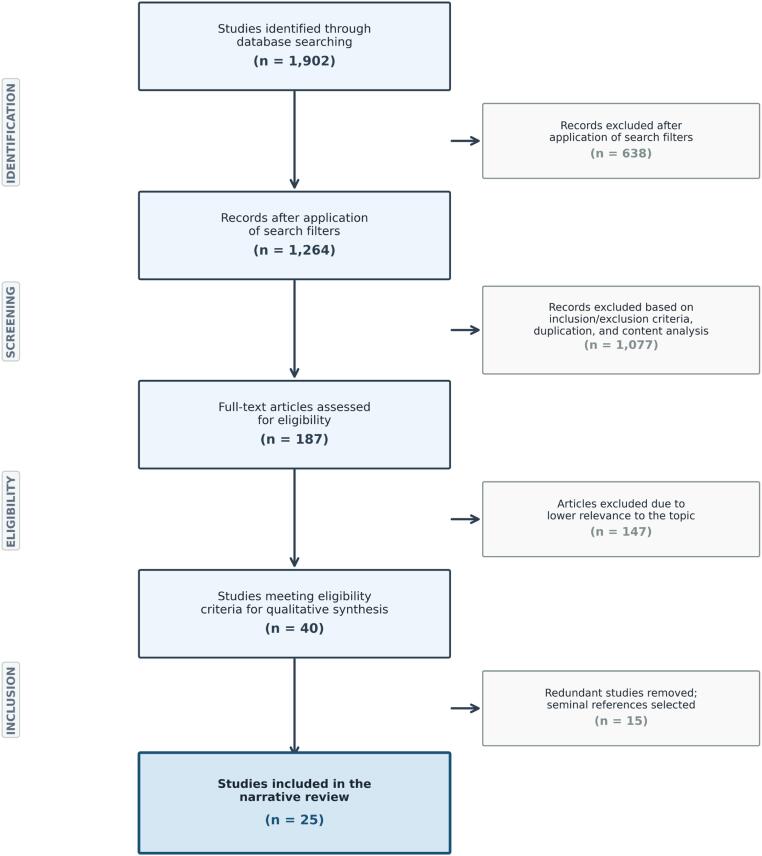
Flow diagram of the study selection process for the narrative review. A total of 1,902 records were identified through electronic database searching. After the application of search filters, 1,264 records remained. Following screening based on predefined inclusion and exclusion criteria, removal of duplicates, and content analysis, 187 full-text articles were assessed for eligibility. Of these, 147 were excluded due to lower relevance to the topic. Among the 40 eligible studies, 15 redundant records were removed, and seminal references were preferentially retained, yielding a final sample of 25 studies included in the qualitative synthesis.

A total of 1,902 articles were identified in the searched databases. Of these, 1,264 articles published between 2014 and 2025 were assessed. Subsequently, 1,077 articles were excluded due to inappropriate or tangential content, lack of relevance, focus on the pediatric population, or unavailability in English, Portuguese, or Spanish. All relevant data were identified, selected, and summarized. Ultimately, 40 highly relevant articles were identified, from which 25 seminal studies were specifically selected to construct this expert review, as illustrated in our flowchart (Figure-1).

## RESULTS

### Definition and Physical Principles

**Figure 2:**
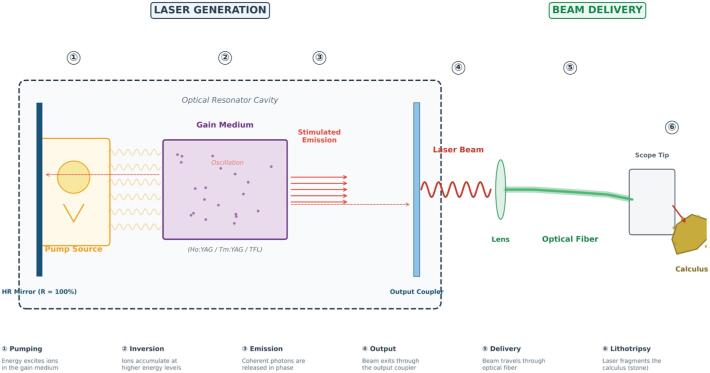
Schematic representation of the fundamental components and operating principles of a solid-state laser system used in urological lithotripsy. The diagram illustrates six sequential stages: ① optical pumping, in which energy from a flashlamp or laser diode excites dopant ions (Ho^3+^ or Tm^3+^) within the gain medium (YAG crystal or silica fiber); ② population inversion, whereby excited ions accumulate at higher energy levels; ③ stimulated emission, where incident photons trigger the release of identical, phase-coherent photons; ④ beam output through the partially reflective output coupler of the optical resonator cavity; ⑤ coupling and delivery of the coherent laser beam through a flexible optical fiber (core diameter 200–550 μm); and ⑥ photothermal lithotripsy at the target calculus via ureteroscopy. The high-reflectance (HR) mirror (R ≈ 100%) and the output coupler define the resonator cavity where photon amplification occurs through multiple oscillation cycles. Original figure; created by the authors.

A laser device (Light Amplification by Stimulated Emission of Radiation) generates a highly directed and precise light beam characterized by photon coherence, where all photons share the same phase and direction, enabling stimulated emission (Figure-2). The mechanism by which lasers fragment urinary stones during lithotripsy is primarily photothermal, involving the direct conversion of laser energy into heat within the stone structure. When laser energy is absorbed by stone components, it generates intense localized heating that creates thermal conditions exceeding the molecular stability of stone materials. This thermal process causes decomposition of chemical bonds within the structure, resulting in stone fragmentation with efficiency dependent on the stone's absorption characteristics for the specific laser wavelength used. Additionally, vapor bubbles are generated through cavitation during the negative pressure phase of shock waves. These bubbles collapse violently within microseconds, producing secondary shock waves and microjets. The asymmetric collapse generates transient pressures near the stone surface. Cumulative microstructural damage from repeated cavitation events leads to stone disintegration.

Key laser properties include high intensity energy delivery to specific points, monochromaticity (single wavelength light), and low beam divergence (directionality) for precision applications. Lasers operate in continuous or pulsed modes, where continuous mode delivers energy constantly with gradual tissue heating that increases thermal damage risk, while pulsed mode emits energy in intermittent bursts with intervals allowing heat dissipation. Pulsed delivery is preferred for lithotripsy to minimize heat transfer to adjacent tissues, with duty cycle control being fundamental for maintaining thermal safety during stone fragmentation procedures.

The following are laser-related concepts that have implications in medical practice:

Pulse Frequency: The repetition rate measured in Hertz (Hz), determining how many pulses are emitted per second.

Pulse Duration: The time length of each individual pulse. Shorter pulses generally generate fewer thermal side effects but may increase retropulsion.

Wavelength: The distance between two consecutive electromagnetic wave peaks, measured in nanometers (nm), determining how laser light interacts with different tissues. Each tissue has specific absorption coefficients for different wavelengths, influencing penetration and therapeutic effects.

Peak Power: The maximum instantaneous laser power during pulse emission, measured in watts (W) or kilowatts (kW), calculated by dividing pulse energy by duration. This parameter directly influences tissue ablation capacity and stone fragmentation efficiency, with higher peak power values enabling more efficient fragmentation with lower total energy requirements.

Absorption Rate: The rate at which laser energy is absorbed by target tissue, determined by the tissue's absorption coefficient (μa) at the specific wavelength.

Efficiency: The relationship between useful energy delivered to target tissue and total system energy, considering transmission, scattering, and non-specific absorption losses, measured as ablated volume per energy unit (μm³/μJ). Efficiency is influenced by wavelength, pulse duration, frequency, tissue optical properties, and application technique, with more efficient systems requiring less total energy and reducing collateral thermal damage and operative time.

These operational parameters are selected based on clinical context and surgeon preference.

### Currently Available Laser Types

The Holmium:YAG laser is considered the gold standard for lithotripsy due to its effectiveness in fragmenting all stone types. The laser emits pulses with a duration ranging from 350 to 700 µs, classified as short-pulse (350 µs) or long-pulse (700 µs), and has a wavelength of 2120 nm with a tissue penetration depth of approximately 0.4 mm.

Ho:YAG lasers come in low-power (25-50 W) and high-power (80-120 W) configurations. Low-power systems use a single laser cavity directly aligned with fiber, typically operate at lower pulse energies (1.0 J), and offer cost-effectiveness. High-power systems incorporate multiple laser cavities requiring precise alignment, achieve higher pulse energies (up to 2.0 J) and frequencies (100 Hz), but demand sophisticated cooling systems and specialized electrical installations (e.g., a dedicated 50 A circuit), increasing costs. Both configurations achieve comparable success and complication rates, though high-power systems may reduce operative times for larger stones (2, 8).

The Moses™ technology enhances Holmium efficiency by delivering modulated pulses with two peaks: the first creates a water cavity facilitating more effective transmission of the second peak to the target stone. This reduces retropulsion and improves fragmentation efficiency, potentially shortening procedures. However, studies show no significant differences in stone-free rates and complications compared to conventional mode. In addition, these systems tend to produce higher noise levels and are generally bigger and heavier than their conventional counterparts, which can impact operating room logistics and ergonomics (2, 3).

**Figure 3:**
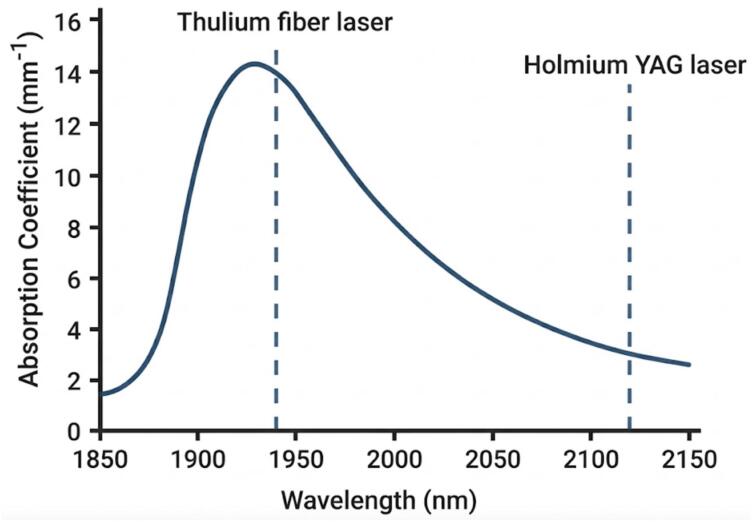
Graph showing the variation of the absorption coefficient of the employed laser according to different wavelengths.

Thulium lasers represent another important advancement in laser technology, comprising two main types: the Thulium Fiber Laser (TFL) and the pulsed Thulium:YAG (Tm:YAG) laser. The Thulium Fiber Laser operates at 1,940 nm, where water absorption of laser energy is higher, exhibiting significantly higher absorption with a coefficient of 160 cm^−1^ (Figure-3). TFL consists of a long 10–20-micron silica fiber doped with Thulium that is characterized by adjustable pulse energies and frequencies, allowing for customization to meet specific clinical needs. Its key advantage lies in achieving high frequencies with lower energy (peak power), highlighting the TFL's potential for a different performance in lithotripsy.

The Thulium:YAG laser emits at 2,013 nm, offering intermediate absorption characteristics (∼100–150 cm^−1^), and features a Thulium crystal in the cavity which is stimulated by high-power laser diodes. Despite its higher peak power compared to the TFL—suggesting potential advantages in stone ablation—its clinical adoption remains limited when compared to Ho:YAG systems and TFL, due to its more recent release (2). The Thulium:YAG system offers various operational modes including Virtual Basket™, Bubble Blast™, and VaporTunnel™, each providing distinct advantages in stone fragmentation protocols.

Optical fibers consist of a high-purity silica core surrounded by a lower refractive index cladding that confines light through total internal reflection, with efficiency determined by the fiber's numerical aperture (NA). They transmit energy from lasers to stones, with 200 or 270 μm fibers being most commonly used in urological lithotripsy practice. Smaller fibers enable better irrigation, reduced retropulsion, and greater endoscopic flexibility. Technical challenges include tip degradation from burnback and curvature radius effects, driving innovations such as detachable tips, automated vibratory fibers, and integrated pressure/temperature sensors to optimize procedure efficacy and safety.

### Safety and Endourological Use

Thermal safety is critical in endourological laser applications. Research demonstrates that proper continuous irrigation and ureteral access sheaths effectively maintain temperatures below tissue-damaging thresholds (2, 3). Appropriate power settings and irrigation rates are essential for thermal injury prevention. Recent ex-vivo and real-world temperature monitoring studies from 2024 and 2025 reaffirm that keeping intrarenal temperatures below the critical 43°C threshold is highly dependent on continuous fluid irrigation (at least 15–20 mL/min), especially when utilizing high-power settings in both Ho:YAG and modern TFL devices (9, 10).

While equipment-related complications occur (e.g., fiber tip degradation), clinically significant adverse events including ureteral perforations and strictures are well documented (2,4), highlighting the importance of precise technique and appropriate laser settings during lithotripsy and soft tissue procedures. Recent evidence reinforces that understanding the nuances of these evolving lithotripsy technologies and tailoring operative parameters are fundamental steps to reduce morbidity and improve the overall safety profile of ureteroscopy (11).

Several techniques can minimize thermal injuries during laser lithotripsy: reducing laser energy/frequency, limiting activation time, increasing irrigation flow, and using room temperature or cooled irrigation fluids (12). Ureteral access sheaths (UAS) facilitate irrigation outflow, reducing intrapelvic pressure and intrarenal temperature—particularly important with high-power laser settings (12). Low-power configurations prevent significant temperature increases and may not require high irrigation rates. Animal studies demonstrate that 15 mL/min irrigation is safe for 25 W lasers, while at least 30 mL/min is necessary for 48 W (13). Dusting strategies correlate with shorter procedures and lower ureteral injury risk, though residual fragments may increase future stone event probability (14).

### Comparative Cost Analysis and System Characteristics

The Holmium:YAG (Ho:YAG) laser is a well-established technology in endourology, widely used for its versatility and efficacy in treating urinary stones. The cost of Holmium laser systems generally ranges from USD 75,000 to USD 350,000, depending on their power output and specific functionalities. In contrast, the Thulium Fiber Laser (TFL) represents a newer option in lithotripsy, featuring a smaller, lighter device that is easier to transport with high efficiency in stone pulverization. These features, however, come at a higher price, with TFL systems typically costing between USD 220,000 and USD 450,000. The Thulium:YAG emerges as an interesting option with great potential for achieving a high peak power, but it is still expensive with values between USD 250,000 to USD 500,000 and remains in early adoption stages. A comprehensive comparison of the technical specifications, advantages, limitations, and costs of these platforms is detailed in Table-1.

**Table 1:** Comparative overview of laser technologies currently available for ureteroscopic lithotripsy.

Category	Parameter	Low Power Ho:YAG	High Power Ho:YAG	Thulium Fiber Laser (TFL)	Thulium YAG (Tm:YAG)
Economic	Estimated cost (USD)	50,000–75,000	150,000–300,000	150,000–300,000	80,000–160,000
Technical Specifications	Wavelength (nm)	2,100	2,100	1,940	2,013
	Peak power (W)	30	> 2,000	< 500	3700
	Pulse frequency (Hz)	5–20	> 120	> 2,000	300
Clinical Performance	Key advantages	Low acquisition cost High energy efficiency	Faster ablation of large/hard calculi Effective across all stone compositions High peak-power capability	Compact, lightweight, portable unit Superior dusting at high frequency Excellent fine fragmentation efficiency	Combines high peak power with pulse modulation Advanced programmable pulse profiles
	Main limitations	Longer operative times for large/hard stones Limited dusting capability	Requires dedicated infrastructure (50 A circuit, cooling) Large, heavy, non-portable unit	Reduced performance on large/hard calculi	Highest acquisition cost Lack of robust long-term comparative data Still in early clinical adoption phase

Ho:YAG = holmium:yttrium-aluminum-garnet; TFL = thulium fiber laser; Tm:YAG = thulium:yttrium-aluminum-garnet; Cost estimates are approximate and may vary by region and vendor

The consumables required for the systems, such as laser fibers, also contribute significantly to the overall cost of treatment. These fibers, available in different diameters, range from USD 200 to USD 1,000 per unit. Most fibers are designed for single use to ensure optimal sterility and performance. Nevertheless, some systems allow for the use of reusable fibers, which, while potentially reducing immediate costs, have a limited number of uses and must be replaced periodically.

### Challenges in Comparative Analysis and Standardization

A critical challenge in interpreting the current literature regarding laser lithotripsy lies in the lack of standardization across studies. The definition of "stone-free rate" (SFR) varies significantly, ranging from the complete absence of fragments to the presence of residual fragments <4mm, <2mm, or even dust, evaluated by different imaging modalities (CT scans vs. Ultrasound vs. KUB). Furthermore, laser settings (frequency, pulse width, and modulation) are often poorly reported or inconsistent between study arms. This heterogeneity makes direct meta-analytical comparisons difficult and highlights the urgent need for a consensus on definitions and reporting standards to solidify the theoretical ground for future comparative research.

### Clinical Decision-Making: A Context-Based Approach

Given the heterogeneity of the literature and the distinct advantages of each technology, the question is not "which is the best laser," but rather "which laser best fits a specific clinical reality." We propose a decision-making framework based on four distinct institutional scenarios:

1. Limited Budget and Low-Complexity Volume: The Low-Power Ho:YAG Solution. For centers with budget constraints or lower surgical volume, the low-power Ho:YAG (20–35W) remains the most rational choice. It is a "workhorse" technology: reliable, cost-effective, and capable of treating the majority of ureteral and renal stones. Although it lacks the speed of high-frequency dusting, its proven efficacy and lower acquisition/maintenance costs offer the best cost-benefit ratio for standard cases.
2. High Performance and Versatility: The High-Power Ho:YAG Solution. For high-volume centers handling complex cases, high-power Ho:YAG systems represent the consolidated gold standard. While requiring significant infrastructure (specialized electrical circuits) and higher capital investment, they offer unmatched versatility for both lithotripsy and enucleation (HoLEP), making them ideal for comprehensive endourology departments.
3. Efficiency and Maximized Dusting: The Thulium Fiber Laser (TFL) Solution. For institutions focused on optimizing operative time and achieving high stone-free rates through dusting, TFL is the superior contender. Its ability to generate ultra-high frequencies allows for the production of fine dust, potentially reducing the need for basket retrieval and shortening procedure times. It is particularly advantageous for soft tissue ablation and precision lithotripsy, representing the modern standard for efficiency.
4. Emerging Technologies and Future Potential: Tm:YAG and Magneto Technology. New entries such as the pulsed Tm:YAG and the hybrid Magneto Cyber Ho represent the frontier of lithotripsy. They offer promising theoretical advantages—such as combining high peak power with TFL-like pulse trains. However, due to their high cost and the current scarcity of high-level comparative evidence, they are currently best suited for academic centers or early adopters aiming to investigate the limits of new lithotripsy modalities.

### Cost-benefit in Relation to Clinical Application: Stone-Free Rate, Complications, Surgical Time

Recent high-quality studies demonstrate that thulium fiber laser (TFL) generally achieves equivalent or superior stone-free rates (SFR) compared to standard Ho:YAG laser in ureteroscopy procedures. A pivotal randomized controlled trial revealed that overall 3-month SFR was significantly higher with TFL at 92% versus 67% with Ho:YAG (p=0.001), with this advantage being particularly pronounced for renal stones (86% vs 49% SFR, p=0.001) (15). Supporting meta-analyses have corroborated this benefit, with one comprehensive pooled analysis of 21 studies (totaling 3598 cases) demonstrating a modest but statistically significant SFR advantage for TFL (RR=1.09, p=0.01) (16).

Regarding safety profiles, overall complication rates remain low and demonstrate broad similarity across laser modalities in ureteroscopy. Pooled analyses have identified no significant differences in total intraoperative or postoperative complications between TFL and Ho:YAG systems (17). Notably, randomized evidence indicates fewer adverse events with TFL, particularly regarding visually impairing bleeding, which occurred significantly less frequently with TFL compared to Ho:YAG (5% vs 22% of cases, p=0.014) (15). While major complications like ureteral perforation and sepsis remain consistently low across all modalities, one meta-analysis found higher postoperative urosepsis rates with TFL (RR≈5.3) (16). However, overall evidence supports similar safety profiles for all three laser systems, with TFL potentially reducing intraoperative bleeding and stone retropulsion. These findings have been consistently corroborated by the most recent 2025 randomized controlled trials and systematic reviews, which confirm that TFL not only achieves comparable or higher stone-free rates but also significantly reduces operative and lithotripsy times when compared to conventional Ho:YAG systems (18, 19).

From an operational efficiency perspective, TFL consistently demonstrates shorter ureteroscopy duration compared to Ho:YAG laser. Both clinical trials and meta-analyses report significantly reduced operative and lasing times with TFL implementation (15, 17). One randomized controlled trial documented mean URS time of 49 minutes with TFL versus 57 minutes with Ho:YAG (p=0.008) (15), representing a clinically meaningful reduction in procedural duration. Retrospective cost analyses have translated these time savings into substantial economic benefits, with one institutional series finding that TFL reduced operating room time by approximately 13 minutes per case, equating to roughly $440 in direct OR cost savings at $34 per minute (20). Additionally, institutional costs per disposable fiber favor TFL at approximately $320 compared to $450 for Ho:YAG fibers (20). The aggregate evidence indicates that TFL, and potentially pulsed Thulium:YAG, can match or exceed Ho:YAG efficacy in ureteroscopy while simultaneously reducing operative time and associated healthcare costs. This continuous pursuit of enhanced surgical efficiency and optimized stone-free rates remains a central focus in contemporary endourological research, continuously shaping our clinical decision-making process (21).

### Future Directions: Emerging Hybrid and Solid-State Technologies

The Cyber Ho system with Magneto Technology combines controlled Holmium:YAG power with extended pulse trains similar to TFL. This creates a vapor channel intended to enable fine fragmentation ("dusting") with reduced retropulsion. A recent multicenter RCT (April 2025) evaluated 60 patients (Magneto vs. TFL). Results demonstrated that the Magneto group had significantly shorter lasing time (12.1 vs 17.3 min, p=0.04) and reduced need for basket retrieval (13.3% vs 23.3%, p=0.04), with similar stone-free rates (22). This technology represents a significant evolution, though further studies are needed to confirm cost-effectiveness.

Similarly, the pulsed solid-state Thulium:YAG (p-Tm:YAG) represents a novel category, distinct from both Ho:YAG and TFL. Panthier et al. (23) reported the initial clinical experience with this technology in 25 patients undergoing retrograde intrarenal surgery (RIRS). The study demonstrated that p-Tm:YAG is safe and feasible, achieving effective fragmentation with no major intraoperative complications. The device combines the peak power characteristics of solid-state lasers with the thulium wavelength (2013 nm), theoretically offering a balance between the explosive power of Ho:YAG and the absorption efficiency of TFL. While Panthier et al. confirmed its clinical viability, they highlighted that specific parameter optimizations are still necessary to fully compete with established dusting standards. A subsequent prospective comparison of TFL and p-Tm:YAG during flexible ureteroscopy demonstrated comparable efficiency and safety profiles between the two technologies, reinforcing the clinical applicability of this emerging platform (24). Further comparative studies are required to strictly define its advantages over TFL in high-volume settings. Recent 2025 systematic reviews, including data from the EAU Section of Endourology, emphasize that p-Tm:YAG positions itself as a highly promising tool specifically for ureteroscopy. Preliminary clinical data for ureterolithotripsy demonstrate high stone-free rates and an excellent safety profile during retrograde approaches, owing to its stable pulse and high peak power (25).

## CONCLUSIONS

Laser technology in endourology has evolved from a one-size-fits-all approach to a diverse ecosystem of specialized tools. While TFL and emerging hybrid technologies demonstrate superior physical properties for dusting and speed, the Ho:YAG platform remains a robust standard. We conclude that no single laser is universally superior. The choice depends on the intersection of cost, stone complexity, and surgical volume. Low-power Ho:YAG serves cost-sensitive centers, while TFL and High-Power systems serve performance-driven institutions. Future research must prioritize standardized reporting of laser parameters and outcomes to allow for clearer comparative analyses.

## Data Availability

All data generated or analysed during this study are included in this published article
